# Key genes of electron transfer, the nitrogen cycle and tetracycline removal in bioelectrochemical systems

**DOI:** 10.1186/s13068-023-02430-z

**Published:** 2023-11-16

**Authors:** Xiaodong Zhao, Xiaorui Qin, Xiuqing Jing, Teng Wang, Qingqing Qiao, Xiaojing Li, Pingmei Yan, Yongtao Li

**Affiliations:** 1 College of Biological Sciences and Technology, Taiyuan Normal University, Yuci, 030619 People’s Republic of China; 2 Agro-Environmental Protection Institute, Ministry of Agriculture and Rural Affairs, Key Laboratory of Original Agro-Environmental Pollution Prevention and Control, MARA, Tianjin Key Laboratory of Agro-Environment and Agro-Product Safety, Tianjin, 300191 People’s Republic of China; 3 College of Natural Resources and Environment, South China Agricultural University, Guangzhou, 510642 People’s Republic of China; 4 Department of Life Science, Changzhi University, Changzhi, 046011 People’s Republic of China

**Keywords:** Soil microbial fuel cell, Tetracycline removal, Functional gene, Nitrogen cycle, Electron transfer

## Abstract

**Background:**

Soil microbial fuel cells (MFCs) can remove antibiotics and antibiotic resistance genes (ARGs) simultaneously, but their removal mechanism is unclear. In this study, metagenomic analysis was employed to reveal the functional genes involved in degradation, electron transfer and the nitrogen cycle in the soil MFC.

**Results:**

The results showed that the soil MFC effectively removed tetracycline in the overlapping area of the cathode and anode, which was 64% higher than that of the control. The ARGs abundance increased by 14% after tetracycline was added (54% of the amplified ARGs belonged to efflux pump genes), while the abundance decreased by 17% in the soil MFC. Five potential degraders of tetracycline were identified, especially the species *Phenylobacterium zucineum,* which could secrete the 4-hydroxyacetophenone monooxygenase encoded by EC 1.14.13.84 to catalyse deacylation or decarboxylation. *Bacillus*, *Geobacter*, *Anaerolinea*, *Gemmatirosa kalamazoonesis* and *Steroidobacter denitrificans* since ubiquinone reductase (encoded by EC 1.6.5.3), succinate dehydrogenase (EC 1.3.5.1), Coenzyme Q-cytochrome c reductase (EC 1.10.2.2), cytochrome-c oxidase (EC 1.9.3.1) and electron transfer flavoprotein-ubiquinone oxidoreductase (EC 1.5.5.1) served as complexes I, II, III, IV and ubiquinone, respectively, to accelerate electron transfer. Additionally, nitrogen metabolism-related gene abundance increased by 16% to support the microbial efficacy in the soil MFC, and especially EC 1.7.5.1, and coding the mutual conversion between nitrite and nitrate was obviously improved.

**Conclusions:**

The soil MFC promoted functional bacterial growth, increased functional gene abundance (including nitrogen cycling, electron transfer, and biodegradation), and facilitated antibiotic and ARG removal. Therefore, soil MFCs have expansive prospects in the remediation of antibiotic-contaminated soil. This study provides insight into the biodegradation mechanism at the gene level in soil bioelectrochemical remediation.

**Supplementary Information:**

The online version contains supplementary material available at 10.1186/s13068-023-02430-z.

## Background

The widespread use and sustained release of antibiotics pose an enormous threat to the ecological environment, leading to the occurrence of bacterial resistance through antibiotic resistance genes (ARGs) [1, 2]. Global analysis of 1088 soil metagenomic samples found that hot spots of microbial resistance are mainly located in densely populated areas with developed agriculture and animal husbandry, such as the eastern United States, western Europe, South Asia and East Asia [3]. Moreover, antibiotics have been discovered in children and pregnant women [4, 5], 93% of the elderly are harmed by antibiotics [6], and 12% of newborns are diagnosed with invasive bacterial infections [7], which poses a serious threat to human health.

Microbial fuel cells (MFCs) have been confirmed to remove antibiotics and ARGs in wastewater [8–10] and soils [11–13]. For instance, Xu et al. [14] reported that the removal rate of sulfamethoxazole in wastewater reached 94% using MFC-constructed wetlands. Our previous research showed that 42–50% of tetracycline in soil MFCs could be removed within 7 days, while its degradation rate in control soil was only 6% [13]. Furthermore, the abundance of ARGs in soil MFCs declined by 19–27% compared with the control [13]. However, the heterogeneity of soil leads to difficulties of the MFC in remediation of antibiotic-contaminated soil compared to that of the water environment; therefore, few studies have reported on the removal mechanism of antibiotics and ARGs in soil using MFCs [15].

Microorganisms are important members of the soil system and are crucial in the degradation of pollutants in soils [16]. Soil MFCs can reshape the interspecific relationship of microorganisms and establish a microbial metabolic network with the ability to efficiently degrade antibiotics while also reducing the number of resistant microbes [17]. At present, many potential degraders of antibiotics have been reported, such as *Bacillus* sp., *Shewanella* sp., *Sphingomonas* sp., *Phenylobacterium* sp., and *Paraclostridium* sp. [18–21]. Unfortunately, most microorganisms are uncultivable, which greatly limits the isolation of degrading microbes [22]. It has become a potential pollution remediation strategy to identify functional genes with antibiotic degradation abilities and construct genetically engineered bacteria. However, to our knowledge, few studies have explained the degradation mechanism of antibiotics by soil MFCs based on functional genes, which requires further study.

The shortage of available nitrogen in organic-contaminated soil is the main limiting factor of bioremediation [23]. As is well known, nitrogen availability is closely related to the nitrogen metabolism, whereas it is restrained by antibiotics. For example, sulfadiazine inhibited nitrification functional genes and nitrobacteria in the surface sediment, resulting in NH_4_^+^ and NO_2_^−^ accumulation in overlying water [24]. The presence of oxytetracycline, sulfamethazine, and ciprofloxacin restrained urea decomposition and denitrification by reducing the abundance of functional genes, including *ureC*, *nirK* and *norB*, in soil [25]. Interestingly, soil MFCs promoted cathode-dominated ammoniation and anode-dominated denitrification while degrading petroleum hydrocarbons [26]. However, thus far, it is unclear whether soil MFCs promote nitrogen cycling during antibiotic removal and which key functional genes are involved.

Previous studies showed that the bioelectricity generated by soil MFCs could stimulate the growth of functional microbes, thus promoting the degradation of pollutants [13, 27, 28]. This is mainly because the metabolism of organic matter is accelerated through electron transfer, hence it is necessary to study the electron transfer process in soil MFCs. Bidirectional extracellular electron transfer (EET), namely, outwards EET and inwards EET, is regarded as the key for the electrochemical activity of electrically active bacteria [29]. In soil MFCs, outwards EET normally occurs at the anode, and the cathode serves as a sustained electron donor for electrotrophic bacteria to conduct inwards EET [13]. Zhang et al. [30] found that conjugated polymers improved the bidirectional EET efficiency by the close biointerface interactions of conjugated polymer-microbe biohybrid systems. Riboflavin is also conducive to enhancing bidirectional EET, and its mechanism is determined by the EET direction: for outwards EET, free riboflavin serves as a redox mediator; for inwards EET, bound riboflavin is involved in electricity consumption [31]. Recently, the impact of adding exogenous substances to electron transfer in soil MFCs has received widespread attention. Chen et al. [32] reported that the addition of insulative ferrihydrite in a soil MFC generated more bioelectricity than conductive magnetite, possibly because ferrihydrite was turned into small particles of semiconductive lepidocrocite/goethite, which was likely to promote long-distance electron transfer. Zhang et al. [33] showed that both dissolved (Fe^2+^) and solid-state (Fe_2_O_3_) electron media acted as electron transporters in soil MFCs. Moreover, the possible electron transfer pathways in soil MFCs also need to be further studied.

In this study, tetracycline was selected to study soil MFCs with the following aims: first, to identify potential degrading bacteria and functional genes to reveal the biodegradation mechanism of tetracycline by soil MFCs; second, to explore the electrically active bacteria and functional genes involved in electron transfer in soil MFCs to speculate on probable electron transfer pathways; and third, to reveal functional genes involved in the nitrogen cycle of soil MFCs and their contribution to tetracycline removal.

## Results and discussion

### Reciprocal action of the anode and cathode was more conducive to tetracycline removal

**Fig. 1:**
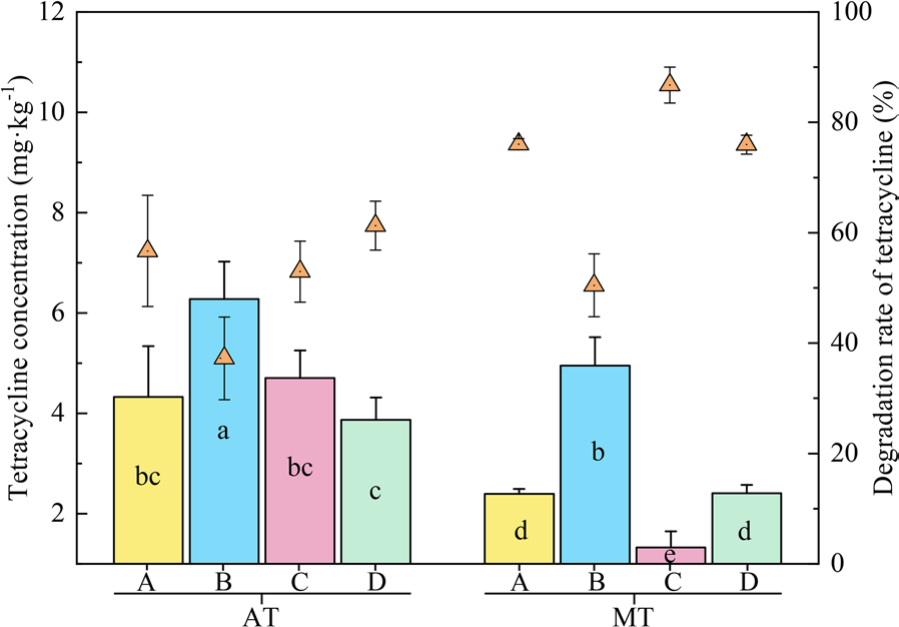
Contents (column graph) and degradation rates (scatter graph) of tetracycline in different treatments. Different lowercase letters represent significant differences at the 0.05 level. The soil MFC and its anaerobic control spiked with tetracycline were labelled MT and AT, respectively

The anode (area A) of the soil MFC treatment (MT) showed superior removal capacity for tetracycline, with a degradation rate of 76%, which increased by 34% (*p* < 0.05) compared with that in the corresponding area of the anaerobic controls spiked with tetracycline (AT) (Fig. 1). To our knowledge, most studies have mainly focused on the degradation mechanism of tetracycline at the MFC anode, while fewer studies have studied the removal mechanism of tetracycline at the cathode. In this study, a similar degradation rate was found between the cathode and anode of the soil MFC, suggesting great potential for the cathode removal of tetracycline [13, 17]. Wang et al. [34] found that electroactive bacteria (*Rhodopseudomonas* sp. and *Acetobacter* sp.) enriched on biocathodes accelerated electron transfer, thereby enhancing the metabolic activity of degraders and ultimately promoting the removal of methyl, hydroxyl, dimethyl, and amide groups on tetracycline. Our previous study also showed that flavoprotein 2,3-oxidoreductase, quinol oxidase and fumarate reductase encoded by EC 1.3.8.7, EC 1.10.3.14 and EC 1.3.5.4, respectively, might promote the electron transfer efficiency from the cathode to the cell, thus strengthening tetracycline removal [13]. Interestingly, in the soil MFC, the highest degradation rate of tetracycline was found in area C (the overlap area of the cathode and anode, 87%), which was 14–72% higher (*p* < 0.05) than other regions of the MFC (Fig. 1). The reason might be that the biodegradation of tetracycline in the C region of the MFC was influenced by both the cathode and anode. In contrast, the tetracycline removal in area B was the lowest but 36% higher (*p* < 0.05) than the corresponding area of the AT treatment.

### Soil bacterial community

#### Anodes dominated the bacterial diversity of the soil MFC

**Fig. 2:**
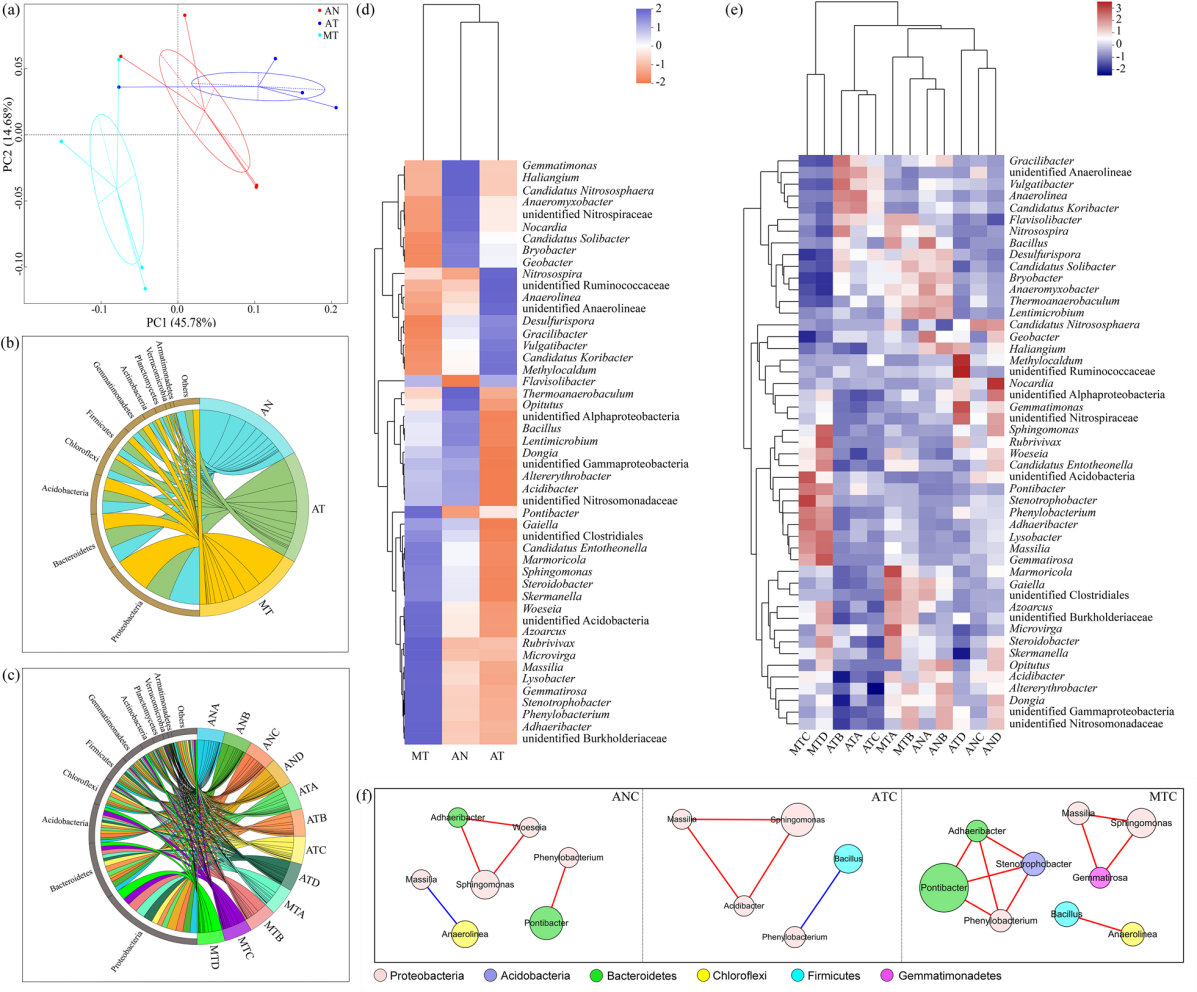
PCoA of the bacterial community (**a**). The anaerobic control without antibiotics was labelled AN. Chord charts of the top 10 bacterial communities at the phylum level in different treatments (**b**) and different areas (**c**). The last capital letter of the sample name represents the sampling area; for example, ANA represents area A of the AN treatment. Heatmaps of the bacterial community at the genus level in different treatments (**d**) and different areas (**e**). Networks of the potential functional bacteria at the genus level (**f**). Red and blue edges represent significant positive and negative relationships, respectively (*p* < 0.05, Spearman test), and the size of each node is proportional to the abundance of the corresponding bacteria

A total of 78,552–78,879 effective tags with average lengths of 254 bp were obtained (Additional file 1: Table S1). The coverage indices were all above 98%, suggesting that the sequencing depths could reflect the real situation of the bacterial community (Additional file 1: Table S1). Compared with the anaerobic controls without antibiotic (AN), the richness declined more obviously (12%) than diversity (4%) in terms of Shannon and Chao1 indices in the AT treatment (Additional file 1: Table S1). In contrast, these two indices in the MT treatment were comparable to those in the AT treatment. In the soil MFC, the highest Shannon index was observed in area A, followed by area C (Additional file 1: Table S2). Furthermore, the Chao1 index of area A was also the highest in the soil MFC, and these among the other three areas were similar. Principal coordinate analysis (PCoA) was conducted for the bacterial community of different treatments based on Bray‒Curtis distances. Axis 1 explained 45.78% of the variance, and Axis 2 explained 14.68% (Fig. 2a). Samples from the AN, AT and MT treatments were clustered together. Furthermore, the distance between MT and AT or AN was farther than that between AT and AN, which indicated that the bacterial community was greatly shifted by the soil MFC.

#### Soil bacterial community composition

At the phylum level, Proteobacteria, Bacteroidetes and Acidobacteria were the top three phyla, with their abundances accounting for 25–37%, 16–19% and 11–13% of the total bacteria, respectively (Fig. 2b). The abundances of Proteobacteria and Acidobacteria in the AT treatment were 18% and 13% lower than those in the AN treatment, respectively. However, the amounts of Proteobacteria and Acidobacteria in the MT treatment increased by 19% and 6% compared with those in the AN treatment, respectively. For Proteobacteria, the highest increase was found in the area B of MT (40%), followed by the area A of MT (34%, Fig. 2c). For Acidobacteria, however, the highest increase was observed in the area C of MT (24%). The abundance of Gemmatimonadetes exhibited a similar trend to that of Acidobacteria.

The abundances of the top fifty genera, accounting for 24–34% of the total composition, were chosen to construct the heatmap (Fig. 2d). The top five genera were *Pontibacter* (2.3–11%), unidentified Acidobacteria (1.4–4.8%), *Anaerolinea* (0.4–5.9%), *Sphingomonas* (1.2–3.7%) and *Bacillus* (0.7–2.2%). The abundance of *Pontibacter* in the AT treatment increased by 49% compared with that in the AN, and it further increased by 64% in the MT treatment (dominant in area C) relative to that in the AT treatment (Fig. 2d, e). Compared with the AN treatment, the abundance of unidentified Acidobacteria and *Sphingomonas* declined by 9% and 22%, respectively, in the AT treatment, whereas they were enhanced by 15% and 14%, respectively, in the MT treatment (Fig. 2d). There were sixteen bacteria showing the same trend as unidentified Acidobacteria and *Sphingomonas*, nine of which belonged to Proteobacteria (*Steroidobacter*, *Phenylobacterium*, *Massilia*, *Azoarcus*, *Microvirga*, *Woeseia*, unidentified Burkholderiaceae, *Skermanella* and *Lysobacter*), one belonged to Acidobacteria (*Stenotrophobacter*), one belonged to Bacteroidetes (*Adhaeribacter*), two belonged to Actinobacteria (*Gaiella* and *Marmoricola*), one belonged to Gemmatimonadetes (*Gemmatirosa*), one belonged to unidentified Bacteria (Candidatus Entotheonella) and one belonged to Firmicutes (unidentified Clostridiales). Unidentified Acidobacteria, *Stenotrophobacter*, *Phenylobacterium* and *Adhaeribacter* were dominant in area C of the MT treatment; *Gemmatirosa* and *Lysobacter* were dominant in areas C and D; *Sphingomonas*, *Massilia*, *Woeseia*, and Candidatus Entotheonella were dominant in area D; *Steroidobacter*, *Azoarcus*, unidentified Burkholderiaceae and *Skermanella* were dominant in areas A and D; *Microvirga*, *Gaiella*, unidentified Clostridiales and *Marmoricola* were dominant in area A (Fig. 2e). In addition, although the amount of *Bacillus* in the AT treatment declined by 21% compared with that in the AN treatment, it rose by 17% in the MT treatment (dominant in area A) relative to that in the AT treatment, as did *Acidibacter*.

### Potential degrading bacteria and electroactive bacteria

The correlation results showed that the degradation rate was significantly positively correlated with *Sphingomonas*, *Stenotrophobacter*, *Phenylobacterium*, *Massilia*, *Adhaeribacter*, *Acidibacter*, *Woeseia* and *Gemmatirosa* (*p* < 0.05, Additional file 1: Table S3). Previous studies indicated that *Sphingomonas* sp. can use tetracycline as a nutrient source for growth and reproduction [18, 19]. *Phenylobacterium* sp. was reported to be capable of degrading sulfonamide antibiotics [35]. *Massilia* sp. and *Gemmatirosa* sp. are potential degrading bacteria of polycyclic aromatic hydrocarbons [36, 37]. *Woeseia* sp. may utilize plastics and hydrocarbons as energy substances [38, 39]. Therefore, the species of these genera might be crucial in tetracycline removal, especially *Sphingomonas* and *Phenylobacterium*.

*Bacillus* sp. and *Geobacter* sp. are potential electroactive bacteria in MFCs [40, 41]. For example, *Bacillus cereus* is capable of promoting electron transfer by aligning the cytochrome complex and excreting flavin molecules [42]. *Geobacter sulfurreducens* can obtain electrons from fumarate and solid donors and conduct extracellular electron transfer through type IV conductive pili and c-type cytochromes [43, 44]. In the current research, the abundance of *Bacillus* in the soil MFC was 17% higher than that in the AT treatment and dominant at the anode. Furthermore, *Geobacter* showed enrichment in the MFC anode. Therefore, *Bacillus* and *Geobacter* were likely to contribute to electricity production. Moreover, the abundance of *Anaerolinea* showed a similar tendency to that of *Geobacter*. *Anaerolinea* sp. was reported to be enriched at the MFC anode and could transfer electrons [45], which might also be a potential exoelectrogen.

The twelve major genera were selected to explore the evolution of interspecific relationships through network analysis (Fig. 2f). Compared with the AN treatment, the numbers of nodes and edges in the network decreased by 29% and 20% in the AT treatment, respectively. This result indicated that the interspecific relationship was weakened after tetracycline was added, which might be attributed to the inhibition of bacteria by tetracycline. In the soil MFC, most of the selected bacteria had a close relationship with each other, and the numbers of nodes and edges were 29–80% and 100–150% higher than those in the other two treatments, respectively (Fig. 2f). This indicated that the interspecific relationship between microorganisms in the soil MFC was strengthened by biocurrent stimulation [17].

### Potential functions of microbiomes in the soil MFC

To identify the potential functions of the soil microbiomes in the MFC, samples from area C (the highest degradation rate in the MT) were chosen for metagenomic sequencing. The clean data were between 6732 and 7183, and the clean Q30 was over 93%, which suggested that the quantity and quality of sequencing data were sufficient (Additional file 1: Table S4). After metagenomic assembly, 186,023–247389 gene fragments were obtained, with N50 lengths reaching 673–958 bp, indicating that the splicing quality was satisfactory for gene prediction.

### Pathway and enzyme-encoding genes of tetracycline biodegradation

**Fig. 3:**
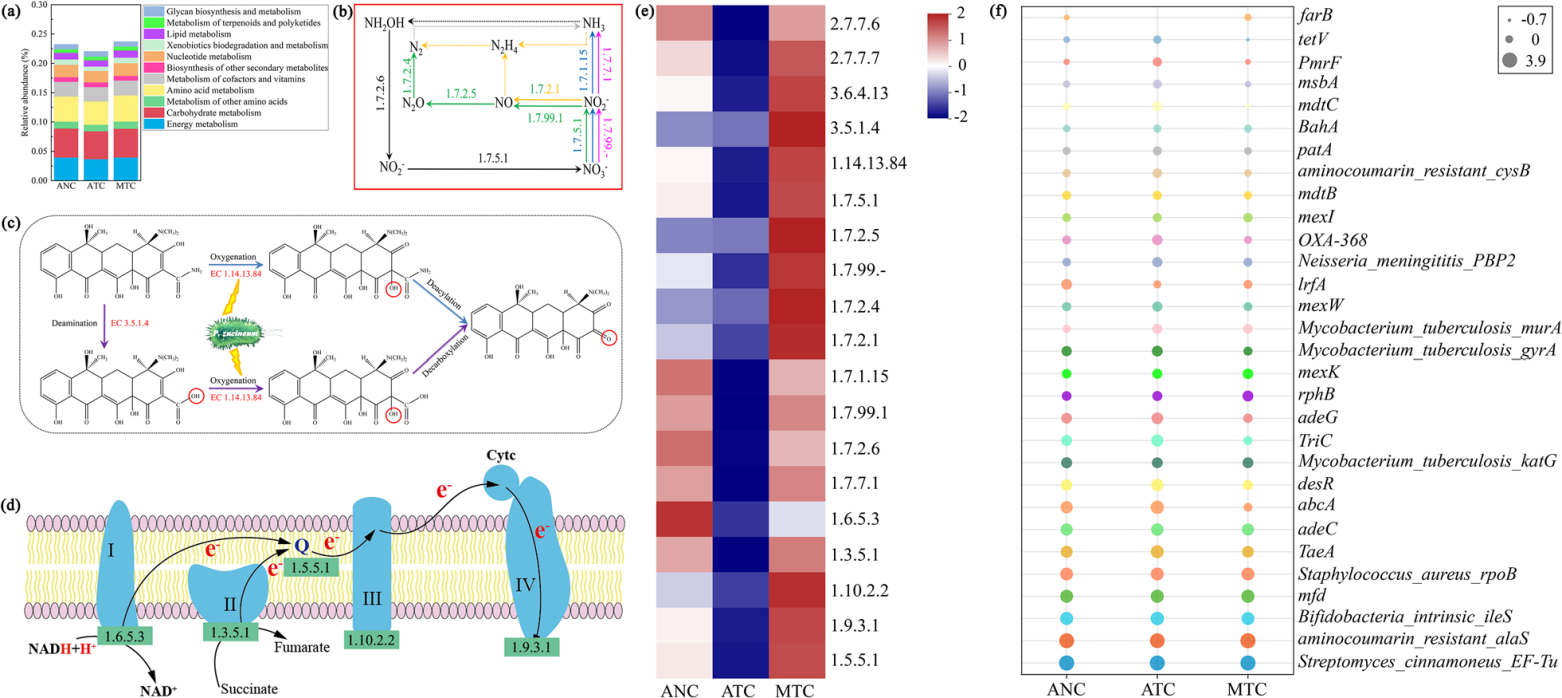
The abundance of metabolism in different treatments (**a**). Proposed nitrogen cycle pathway (**b**), degradation pathway of tetracycline (**c**) and electron transfer pathway (**d**) in the soil MFC. The abundances of enzyme-encoding genes (**e**) and ARGs (**f**) in different treatments

Biological metabolic pathways in the Kyoto Encyclopedia of Genes and Genomes (KEGG) database mostly included energy metabolism, carbohydrate metabolism, amino acid metabolism, metabolism of other amino acids, metabolism of cofactors and vitamins, nucleotide metabolism, xenobiotic biodegradation and metabolism, metabolism of terpenoids and polyketides, and lipid metabolism. In this study, the relative abundance of metabolic pathways in the area C of AT (ATC) declined by 5.1% compared with that in the area C of AN (ANC), whereas it was enhanced by 7.6% in the area C of MT (MTC) compared with ATC, suggesting that soil MFC could restore the metabolism inhibited by tetracycline (Fig. 3a). Especially for xenobiotic biodegradation and metabolism, its abundance in MTC was 25% higher than that in ATC, which reflected the superior degradation performance of MFC. In addition, DNA polymerase (encoded by EC 2.7.7.7, EC 2.7.7.6) and RNA helicase (EC 3.6.4.13) abundances in the MTC treatment were enhanced by 16–27% and 42%, respectively, compared with those in the ATC treatment (Fig. 3e), suggesting that MFC could promote microorganism growth, which confirmed a previous conjecture [13, 26, 46].

Previous studies have shown that the biodegradation pathway of tetracycline mostly included oxidation, demethylation, decarbonylation, dehydrogenation, deamination, dehydroxylation, loss of acyl-amino groups (deacylation) and ring opening, and the degraders included *Sphingobacterium changzhouense* TC931, *Sphingobacterium mizutaii* S121, *Klebsiella* sp. SQY5 and *Alcaligenes* sp. R3 [47–51]. The biodegradation of tetracycline is achieved by complicated metabolic reactions catalysed by enzymes. For example, Yin et al. [52] found that isomerase-, oxidoreductase-, and transferase-encoding genes were possibly involved in tetracycline degradation. In the present study, amidase (EC 3.5.1.4) and 4-hydroxyacetophenone monooxygenase (EC 1.14.13.84) were found to have potential degradation functions. Although the abundance of EC 3.5.1.4 was similar in the ANC and ATC, its abundance in the MTC was 31% higher than that in the ATC (Fig. 3e). Compared with that of the ANC, the abundance of EC 1.14.13.84 decreased by 67% in the ATC, whereas it increased by 59% in the MTC. According to the KEGG database, EC 3.5.1.4 is involved in the degradation of aminobenzoate (reaction: R05590, pathway: map00627, Additional file 1: Figure S1), and it mainly promotes the removal of amino groups [53, 54]. Therefore, the enzyme-encoding gene EC 3.5.1.4 was likely to catalyse the deamination reaction of tetracycline biodegradation in this study (Fig. 3c). Zhou et al. [55] reported that the –NH_2_ in sulfamethoxazole easily underwent a substitution reaction with –OH. Moreover, EC 1.14.13.84 can insert oxygen atoms between the aromatic ring and a ketone side chain to degrade bisphenol (reaction: R06892, pathway: map00363, Additional file 1: Figure S2) [56]. Thus, the EC 1.14.13.84 gene might be involved in the deacylation of tetracycline degradation or act on the bond breaking of the –COOH group (Fig. 3c).

### Nitrogen cycle processes were enhanced by the soil MFC

Nitrogen limitation, as the bottleneck of soil bioremediation, needs attention [57]. Our results showed that the abundance of nitrogen metabolism (ko00910) in the ANC decreased by 11% compared with that in the ATC (Additional file 1: Figure S3). Previous studies showed that tetracyclines might inhibit nitrification, denitrification and anammox processes, and the rates of nitrification, denitrification and anammox activity declined by 50%, 44% and 81% relative to nonantibiotic treatment, respectively [58–60]. However, whether tetracyclines inhibit the nitrogen cycle process is related to the concentration of tetracyclines and the duration of the experiment [61]. Interestingly, the gene abundance of nitrogen metabolism increased by 16% in the soil MFC compared with ATC (Additional file 1: Figure S3). The possible reasons were as follows: first, the electrochemically active microbes enriched in the MFC promoted the geochemical cycle (including the carbon cycle, nitrogen cycle, sulfur cycle, iron cycle, etc.) [62]; second, the growth and metabolism of nitrogen cycle microorganisms might be activated in MFCs [26]; third, the MFC enhanced the shedding of nitrogen-containing groups (amino and amide groups) of tetracycline, thus providing a more abundant nitrogen source for nitrogen cycle microbes.

The nitrogen cycle mostly includes dissimilatory nitrate reduction, assimilatory nitrate reduction, denitrification, nitrification and anammox [63, 64], and enzymes are vital driving factors in nitrogen transformation processes [65]. Nine enzyme-encoding genes related to the nitrogen cycle were identified (Fig. 3b), of which EC 1.7.5.1 showed the highest abundance, followed by EC 1.7.2.5, EC 1.7.99.-, EC 1.7.2.4, EC 1.7.2.1, EC 1.7.1.15, EC 1.7.99.1, EC 1.7.2.6 and EC 1.7.7.1 (Additional file 1: Figure S4). For dissimilatory nitrate reduction, the nitrate reductase encoding EC 1.7.5.1 catalyses the conversion of nitrate to nitrite [66], and the presence of nitrite reductase encoding EC 1.7.1.15 is beneficial for the formation of ammonia [67]. The enzyme-encoding gene EC 1.7.5.1 simultaneously participates in nitrification (nitrite → nitrate) [68]. In addition, the reaction from hydroxylamine to nitrite in nitrification was equally important and was catalysed by hydroxylamine dehydrogenase (EC 1.7.2.6) [69]. The assimilatory nitrate reduction process mostly involves assimilatory nitrate reductase encoded by EC 1.7.99.- and ferredoxin-nitrite reductase encoded by EC 1.7.7.1 [70]. Denitrification was divided into four steps, namely, nitrate → nitrite → nitric oxide → nitrous oxide → nitrogen. Nitrite reductase (EC 1.7.2.1) and hydroxylamine reductase (EC 1.7.99.1) jointly promote the reaction from nitrite to nitric oxide [23, 71]. Nitric oxide reductase (EC 1.7.2.5) and nitrous oxide reductase (EC 1.7.2.4) catalyse the conversion of nitric oxide to nitrous oxide and nitrous oxide to nitrogen, respectively [72]. As expected, the nine enzyme-encoding genes mentioned above were inhibited by tetracycline; however, their abundances were upregulated in the soil MFC, with EC 1.7.5.1 showing the largest increase in abundance, an increase of 79% in the MTC treatment compared to the ATC treatment (Fig. 3e). This result indicated that MFC promoted the nitrogen cycle in soil, especially the mutual conversion between nitrite and nitrate (NO_2_^−^NO_3_^−^) driven by the enzyme-encoding gene EC 1.7.5.1 (Fig. 3b). This meant that there were sufficient electron receptors (NO_3_^−^) and donors (NO_2_^−^), as electron shuttles in the overlapping area of the cathode and anode, which might serve tetracycline degradation.

### Genes of electron transfer were upregulated by the soil MFC

Complexes (I, II, III, IV) containing electronic carriers are crucial in the respiratory chain, which participates in inwards and outwards electron transfer [73]. For instance, our previous research found that the succinate dehydrogenase complex encoded by EC 1.3.5.4 (EC 1.3.5.1) catalysed the conversion of succinate to fumarate at the cathode [13]. A total of four related enzyme-encoding genes (including EC 1.6.5.3, EC 1.3.5.1, EC 1.10.2.2 and EC 1.9.3.1) were discovered in this study. Although their abundances in the ATC declined by 11–34% relative to the ANC, they increased by 20–36% in the MTC compared with the ATC (Fig. 3e). EC 1.6.5.3 encodes ubiquinone reductase (complex I), which is a very large complex that participates in the electron transfer chains of mitochondria and aerobic bacteria, transferring electrons from NADH to the ubiquinone pool [74]. Coenzyme Q-cytochrome c reductase encoded by EC 1.10.2.2 could act as complex III for electron transfer [75]. EC 1.9.3.1 encoded cytochrome-c oxidase that acted on a haem group of donors and could serve as complex IV [75]. In addition, as a liposoluble coenzyme, ubiquinone could accept electrons transferred from complex I or complex II in the respiratory chain and then transfer the electrons to complex III [76]. In the current study, the abundance of electron transfer flavoprotein-ubiquinone oxidoreductase encoded by EC 1.5.5.1 in the ATC treatment declined by 21% compared with that in the ANC treatment, whereas it was enhanced by 44% in the MTC treatment compared with that in the ATC treatment (Fig. 3e). Based on the above analysis, a schematic diagram of intracellular electron transfer is presented in soil MFC (Fig. 3d). Ultimately, the electrons transmitted within the cell are transferred to the electrode through direct contact or electron mediators, thereby accelerating the electron transfer of the soil MFC, which was beneficial for tetracycline degradation.

### ARGs were reduced efficiently by the soil MFC

The thirty most abundant ARGs were selected to draw the bubble chart (Fig. 3f). The total abundance in the ATC increased by 14% compared with that in the ANC; however, it decreased by 17% in the MTC relative to that in the ATC (Additional file 1: Figure S5), indicating that MFC could effectively remove ARGs [13, 17]. Among these thirty ARGs, a total of twenty-five ARGs (Excluding Streptomyces_cinnamoneus_EF-Tu, aminocoumarin_resistant_alaS, Mycobacterium_tuberculosis_katG, *lrfA* and *farB*) increased in the ATC compared with the ANC, with an average increase of 60% (Fig. 3f). In the twenty-five ARGs, 54% belonged to efflux pump genes, 33% belonged to target alteration genes, and 13% belonged to inactivation genes (Additional file 1: Figure S6a). Therefore, efflux pump resistance was the main resistance mechanism of soil microorganisms to tetracycline. The efflux pump, as a transport protein present on the cell membrane, can pump antibiotics out of the cells, thereby reducing the concentration of intracellular antibiotics and leading to bacterial resistance [77, 78]. Furthermore, the efflux pump is the vital cross-resistance mechanism; that is, different pollutants (such as tetracycline or heavy metals) attack the same target and activate the bacterial efflux pump system, causing it to be resistant to multiple pollutants [79]. In this study, the addition of tetracycline not only increased the abundance of tetracycline resistance genes but also amplified other ARGs after tetracycline exposure. For example, among the efflux pump genes, only *adeC*, *adeG*, *mexW*, *mexI* and *tetV* belonged to tetracycline resistance genes. The other efflux pump genes were as follows: *abcA*, peptide antibiotic; *penam*, cephalosporin resistance gene; *TaeA*, pleuromutilin resistance gene; *mdtB* and *mdtC*, aminocoumarin resistance genes; *patA*, fluoroquinolone resistance gene; and *msbA*, nitroimidazole resistance gene. Zhang et al. [80] also found that the abundances of *sulII* and *bla*_*TEM-1*_ were elevated by tetracycline, indicating that tetracycline might function as a coselection pressure for ARGs corresponding to other antibiotics. In addition, nineteen of the thirty ARGs (Streptomyces_cinnamoneus_EF-Tu, Bifidobacteria_intrinsic_ileS, *abcA*, *TaeA*, *desR*, *TriC*, *adeG*, Mycobacterium_tuberculosis_katG, Mycobacterium_tuberculosis_gyrA, *lrfA*, OXA-368, *mexW*, aminocoumarin_resistant_cysB, *mdtC*, *PmrF*, *mdtB*, *patA*, *msbA* and *tetV*) in the MTC treatment were reduced compared with those in the ANC treatment, and the abundance of the nineteen ARGs mentioned above in the MTC treatment was 33% lower on average than that in the ANC (Fig. 3f). Similarly, the efflux pump gene accounted for most ARGs (61%), followed by the target alteration gene (33%) (Additional file 1: Figure S6b).

### Functional microbes at the species level were discovered in the soil MFC

**Fig. 4:**
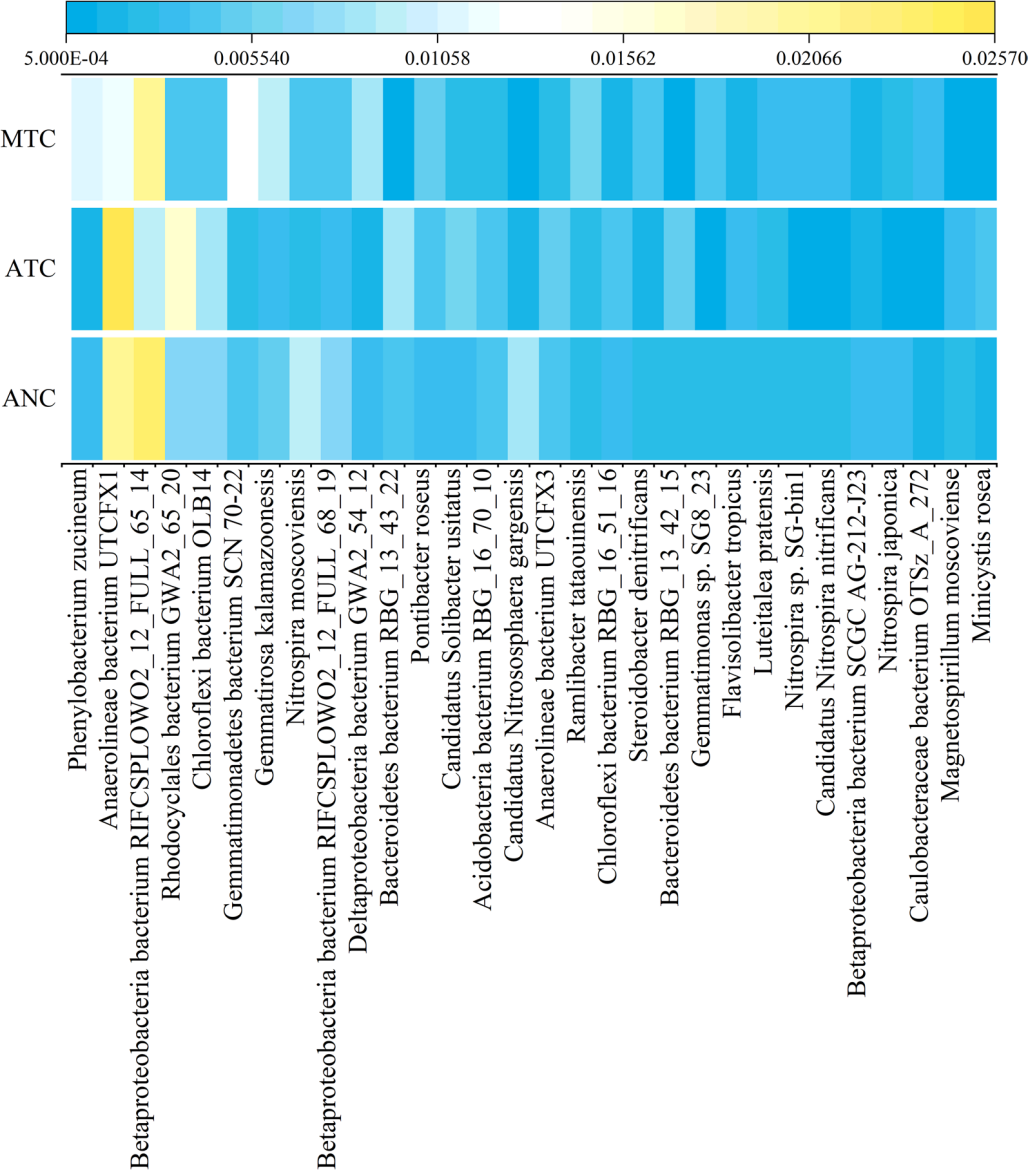
Split heatmap of the microbial community at the species level in different treatments

The abundances of the top thirty species were chosen to cluster the split heatmap (Fig. 4). *Phenylobacterium zucineum* was the top species. Compared with ANC, the amount of *P*. *zucineum* in the ATC treatment decreased by 52%, whereas it was 637% higher in MTC than in ATC (Fig. 4). A total of fourteen species exhibited the same trend as *P*. *zucineum*, such as *Gemmatirosa kalamazoonesis*, *Nitrospira moscoviensis*, *Ramlibacter tataouinensis*, *Steroidobacter denitrificans*, *Nitrospira* sp. SG-bin1, *Candidatus Nitrospira nitrificans* and *Nitrospira japonica*. Furthermore, the abundances of *Pontibacter roseus* and *Luteitalea pratensis* in the ATC, respectively, increased by 18% and 1.2% compared with the ANC treatment, and they rose by 10% and 51% in the MTC treatment relative to the ATC treatment, respectively (Fig. 4).

Chen et al. [35] found that *Phenylobacterium* sp. was a degrader of sulfadiazine and sulfamethoxazole. Interestingly, EC 1.14.13.84 was found in the species *P*. *zucineum*, which suggested that *P*. *zucineum* was likely to secrete 4-hydroxyacetophenone monooxygenase to degrade tetracycline (Fig. 3c). Our previous study indicated that the species* G*. *kalamazoonesis* and *S*. *denitrificans* were potential electrotrophic microbes; *G*. *kalamazoonesis* and *S. denitrificans* secreted quinoloxidase encoded by EC 1.10.3.14 and flavoprotein 2,3-oxidoreductase encoded by EC 1.3.8.7 to accelerate electron transfer, respectively [13]. In the current study, EC 1.6.5.3 and EC 1.9.3.1 were found in both *G*. *kalamazoonesis* and *S. denitrificans*. In addition, EC 1.3.5.1 and EC 1.10.2.2 existed in *G*. *kalamazoonesis* and *S. denitrificans*, respectively. *G*. *kalamazoonosis* and *S*. *denitrificans* were likely to be electrotrophic bacteria and contributed to electricity generation in this experiment. Previous studies showed that the species *N*. *moscoviensis*, *Nitrospira* sp. SG-bin1, *Candidatus Nitrospira nitrificans* and *N*. *japonica* are involved in the nitrogen cycle [81–84]. For instance, *Candidatus Nitrospira nitrificans* could fully oxidize ammonia via nitrite to nitrate [83]. However, unfortunately, no association was found between these species and the nitrogen cycle genes mentioned above.

## Conclusions

Tetracycline and ARGs were effectively removed simultaneously by the soil MFC, which was due to the enrichment of degrading bacteria and electroactive bacteria and their close interactions. Based on the metagenomic analysis,* G*. *kalamazoonesis* and *S*. *denitrificans* are likely to be electrotrophic bacteria, while *P*. *zucineum* can secrete the 4-hydroxyacetophenone monooxygenase encoded by EC 1.14.13.84 to catalyse the deacylation or decarboxylation of tetracycline. Substantially, the soil MFC enhanced microbial metabolism, especially xenobiotic biodegradation and nitrogen metabolism, and corresponding functional genes, including degradation, electron transfer and the nitrogen cycle. In addition, efflux pump resistance, as the main resistance of microbes to tetracycline, was reduced by the soil MFC. Overall, the key functional genes in soil bioelectrochemical remediation were revealed in this study.

## Material and methods

### Tested soils and chemicals

The tested soil sample was collected from Wuqing farmland in Tianjin (coordinate: N39°27′20.59″, E117°09′26.18″) and then air-dried, ground, and passed through a 2-mm sieve. The soil properties are shown in Additional file 1: Table S5. Tetracycline was purchased from Dr. Ehrenstorfer LGC (Augsburg, Germany). Chemicals such as methanol, acetonitrile and acetone were of chromatography grade.

### Soil MFC configuration and operation

**Fig. 5:**
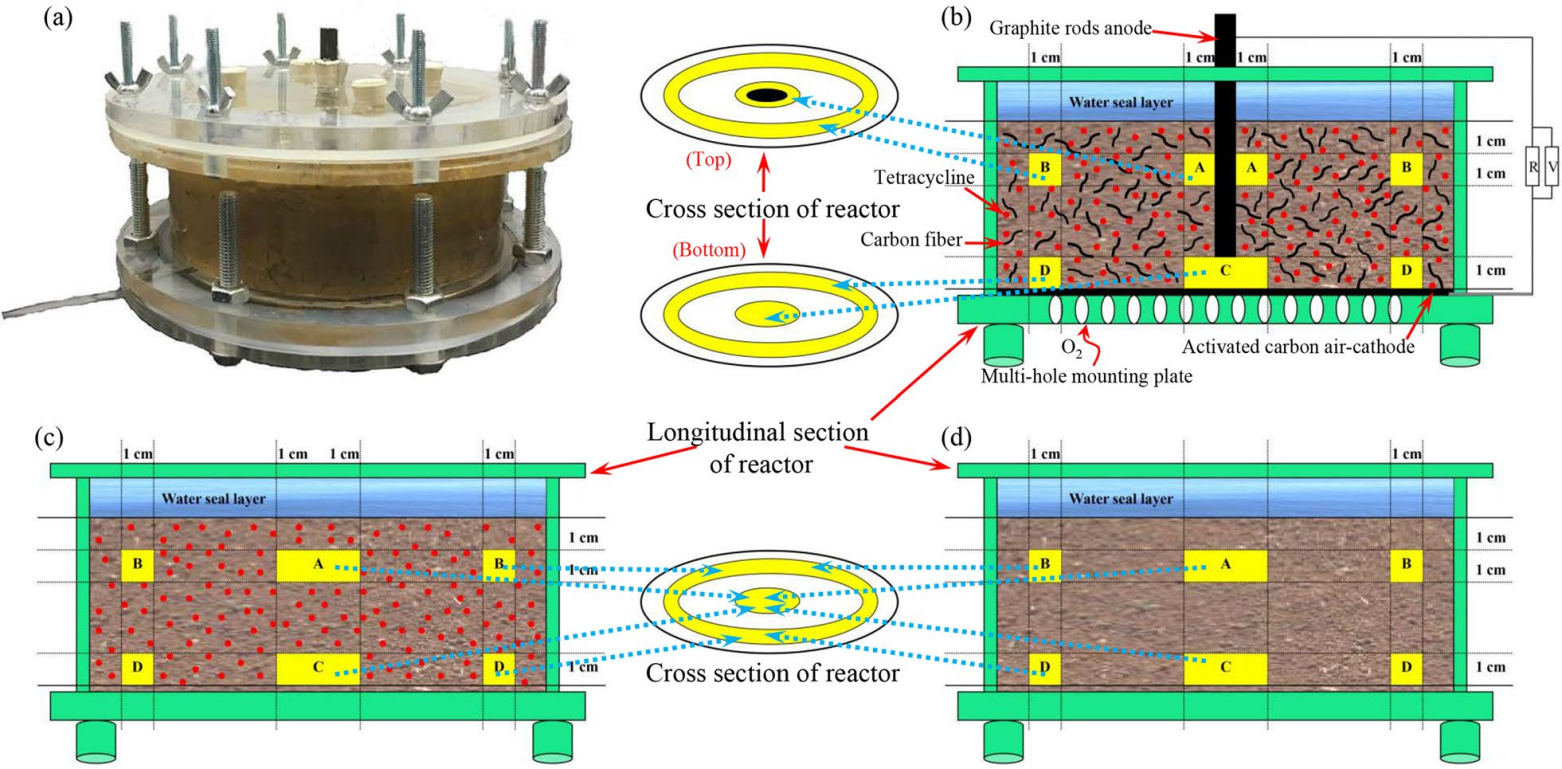
Picture (**a**), schematic drawing of the soil MFC (**b**), open-circuit group (**c**) and nonelectrode group (**d**). The yellow area is the sampling area

The configuration and operation of the soil MFC were performed according to our previous method [85]. Briefly, the cylindrical reactor was composed of a graphite rod anode and an activated carbon air–cathode (Fig. 5). Each reactor was filled with 1000 g of soil (400 mL deionized water, 10 mg·kg^−1^ tetracycline) and connected to an external resistance of 100 Ω (labelled MT). Carbon fibre was mixed into the soil at a 1% mass fraction to reduce soil internal resistance [28]. Furthermore, nonelectrode controls (spiked with 10 mg·kg^−1^ tetracycline, AT; no antibiotic added, AN) were set. The experimental details are shown in the Supplementary Information (Additional file 1: Table S6). All reactors were placed at a constant temperature of 30 °C for 53 days without light.

### Electrochemical and chemical analysis

Voltage (*U*) was recorded using a data acquisition system (PISO-813, ICP DAS Co., Ltd) [13]. The soil samples were collected at the anode (area A), away from the cathode and anode (area B), overlapping area of the cathode and anode (area C), cathode (area D) of the soil MFC, and the corresponding control areas (Fig. 5). The partial samples were stored at − 80 °C for biological analysis. Others were freeze-dried for soil property analysis and tetracycline quantification. The soil pH and electrical conductivity were measured by a metre at a 1:5 soil:water ratio [86]. The total nitrogen, phosphorus and organic matter were analysed by common methods [87]. The tetracycline content was determined by a published protocol [88].

### Analysis of 16S rRNA gene amplicons

Total genomic DNA was extracted from soil samples by a Power Soil DNA isolation kit (Mo Bio, America). The concentration and purity of DNA were determined in 1% agarose gels and then diluted to 1 ng·μL^−1^ with sterile water. The universal primers 515F (GTGCCAGCMGCCGCGGTAA) and 806R (GGACTACHVGGGTWTCTAAT) were used to amplify the V3-V4 regions of the 16S rRNA gene by PCR. The PCR conditions referred to our previous method [13]: 3 min at 95 °C (initial denaturation); 30 cycles consisting of denaturation at 95 °C for 30 s, renaturation at 55 °C for 30 s, and extension at 72 °C for 45 s; and a final extension at 72 °C for 10 min. The samples were assessed in 2% agarose gels by electrophoresis. After purification, a high-quality sequencing library was constructed and sequenced by Novogene Company (Beijing, China).

### Metagenomic analysis

The extracted DNA samples were sequenced on the Illumina NovaSeq 6000 platform (Illumina, San Diego, CA, USA) according to our previous report [13]. Briefly, the genomic DNA was randomly sheared into fragments using a Covaris S2 System (Massachusetts, USA) for library construction. Libraries were quantified using real-time PCR and then sequenced by Novogene Company. Subsequently, the quality of the raw data was filtered to acquire reliable data for assembly. Open reading frame prediction was performed based on each sample and the mixed assembled scaffolds (≥ 500 bp), and reads with a length less than 100 nt were filtered out. CD-HIT software was used to remove redundancy to obtain a nonredundant initial gene catalogue. The clean data of each sample were compared to the initial gene catalogue to obtain the number of gene reads. DIAMOND software was employed to compare the gene catalogue with the sequences of bacteria, fungi, archaea and viruses extracted from the National Center for Biotechnology Information (NCBI) database to obtain species information. Enzyme genes were annotated by the KEGG database, and ARGs were annotated by the Comprehensive Antibiotic Research Database (CARD) database.

### Statistical analysis

Microsoft Excel 2010 (Redmond, USA) was employed to acquire averages and standard deviations of the data. The significant differences and Spearman correlation between samples were determined by IBM SPSS Statistics 22 software (New York, USA). Networks were constructed to reveal the interspecific relationships between the potential functional bacteria at the genus level using Cytoscape 3.9.1 software (California, USA). To reduce the network complexity, Spearman correlation analysis was conducted between functional bacteria, and a correlation between two bacteria was regarded as statistically robust if *p* < 0.05. Nodes represented functional bacteria, and edges represented the interaction between these bacteria.

### Supplementary Information

**Additional file 1: Table S1.** HiSeq sequencing data and alpha indices of the groups. **Table S2.** HiSeq sequencing data and alpha indices of samples. **Table S3.** Spearman correlation between tetracycline degradation rate and bacterial abundance at the genus level. **Table S4.** HiSeq sequencing data of the metagenome. **Table S5.** Physicochemical properties of the experimental soil. **Table S6.** Experimental design. **Figure S1.** Sketch map of KEGG metabolic pathways and related reaction equations (pathway: map00627, reaction: R05590). **Figure S2.** Sketch map of KEGG metabolic pathways and related reaction equations (pathway: map00363, reaction: R06892). **Figure S3.** The abundance of nitrogen metabolism in different treatments. **Figure S4.** The abundance of nitrogen cycling functional genes. **Figure S5.** Changes in the total abundance of ARGs in different treatments. **Figure S6.** The resistance mechanism of soil microorganisms to tetracycline in the ATC treatment (**a**) and the MTC treatment (**b**).

## Data Availability

All data generated or analyzed during this study are included in this published article and its supplementary information files.

## Abbreviations

MFCs: Microbial fuel cells
ARGs: Antibiotic resistance genes
MT: Soil MFC spiked with tetracycline
MTC: The area C of MT
AT: Anaerobic controls spiked with tetracycline
ATC: The area C of AT
AN: Anaerobic controls without antibiotic
ANC: The area C of AN
NCBI: National Center for Biotechnology Information
KEGG: Kyoto Encyclopedia of Genes and Genomes
CARD: Comprehensive Antibiotic Research Database
