# Supplementary material for: Key genes of electron transfer, the nitrogen cycle and tetracycline removal in bioelectrochemical systems
Source: Biotechnol Biofuels Bioprod. 2023 Nov 16;16:174. doi: 10.1186/s13068-023-02430-z (PMC10652473; doi:10.1186/s13068-023-02430-z)
Supplement: Supplementary file 1 — Additional file 1: Table S1. HiSeq sequencing data and alpha indices of the groups. Table S2. HiSeq sequencing data and alpha indices of samples. Table S3. Spearman correlation between tetracycline degradation rate and bacterial abundance at the genus level. Table S4. HiSeq sequencing data of the metagenome. Table S5. Physicochemical properties of the experimental soil. Table S6. Experimental design. Figure S1. Sketch map of KEGG metabolic pathways and related reaction equations (pathway: map00627, reaction: R05590). Figure S2. Sketch map of KEGG metabolic pathways and related reaction equations (pathway: map00363, reaction: R06892). Figure S3. The abundance of nitrogen metabolism in different treatments. Figure S4. The abundance of nitrogen cycling functional genes. Figure S5. Changes in the total abundance of ARGs in different treatments. Figure S6. The resistance mechanism of soil microorganisms to tetracycline in the ATC treatment (a) and the MTC treatment (b). [file 13068_2023_2430_MOESM1_ESM.doc]

***Additional file***

**Key genes of** **electron transfer, the nitrogen cycle and tetracycline removal in bioelectrochemical systems**

Xiaodong Zhao ^1^, Xiaorui Qin ^1^, Xiuqing Jing ^1^, Teng Wang ^4^, Qingqing Qiao ^1^, Xiaojing Li ^2^,*, Pingmei Yan ^1^, Yongtao Li ^3^

^1^ College of Biological Sciences and Technology, Taiyuan Normal University, Yuci 030619, P.R.China

^2^ Agro-Environmental Protection Institute, Ministry of Agriculture and Rural Affairs / Key Laboratory of Original Agro-Environmental Pollution Prevention and Control, MARA / Tianjin Key Laboratory of Agro-Environment and Agro-Product Safety, Tianjin 300191, P.R.China

^3^ College of Natural Resources and Environment, South China Agricultural University, Guangzhou 510642, P.R.China

^4^ Department of Life Science, Changzhi University, Changzhi 046011, P.R.China

* Corresponding Authors: Phone: (86)22-23611021; Fax: (86)22-23613820;

E-mail: lixiaojing@caas.cn (Li X.)

**Table S1** HiSeq sequencing data and alpha indices of the groups.

| Sample name | Effective tags(#) | AvgLen(nt) | Goods coverage | Shannon | Chao1 |
| --- | --- | --- | --- | --- | --- |
| AN | 78552 | 254 | 0.984 | 10.029 | 5485 |
| AT | 78879 | 253 | 0.986 | 9.607 | 4830 |
| MT | 78832 | 254 | 0.985 | 9.484 | 4944 |

Soil MFC spiked with tetracycline was labelled MT, and anaerobic controls spiked with tetracycline and without antibiotics were labelled AT and AN, respectively, the same below.

**Table S2** HiSeq sequencing data and alpha indices of samples.

| Sample name | Effective tags(#) | AvgLen(nt) | Goods coverage | Shannon | Chao1 |
| --- | --- | --- | --- | --- | --- |
| ANA | 73278 | 253 | 0.985 | 9.980 | 5430 |
| ANB | 74297 | 254 | 0.985 | 9.762 | 5393 |
| ANC | 81346 | 253 | 0.984 | 10.162 | 5551 |
| AND | 85287 | 254 | 0.983 | 10.211 | 5566 |
| ATA | 75732 | 254 | 0.986 | 9.662 | 4772 |
| ATB | 75969 | 253 | 0.987 | 9.384 | 4554 |
| ATC | 83162 | 253 | 0.985 | 9.488 | 4964 |
| ATD | 80651 | 253 | 0.985 | 9.892 | 5031 |
| MTA | 80660 | 254 | 0.985 | 9.695 | 5100 |
| MTB | 75478 | 253 | 0.985 | 9.335 | 4969 |
| MTC | 75841 | 253 | 0.985 | 9.515 | 4869 |
| MTD | 83349 | 254 | 0.985 | 9.389 | 4838 |

The last capital letter of the sample name represents the sampling area, for example, ANA represents area A of the AN treatment, the same below.

**Table S3** Spearman correlation between tetracycline degradation rate and bacterial abundance at the genus level.

|  | The degradation rate of tetracycline | | |
| --- | --- | --- | --- |
|  | *r* | *p* | *n* |
| *Pontibacter* | .571 | .139 | 8 |
| unidentified_Acidobacteria | .524 | .183 | 8 |
| *Anaerolinea* | -.595 | .120 | 8 |
| *Sphingomonas* | .738^*^ | .037 | 8 |
| *Bacillus* | -.214 | .610 | 8 |
| *Flavisolibacter* | -.395 | .333 | 8 |
| *Bryobacter* | -.743^*^ | .035 | 8 |
| *Gemmatimonas* | .500 | .207 | 8 |
| unidentified_Gammaproteobacteria | .310 | .456 | 8 |
| Candidatus_Solibacter | -.659 | .076 | 8 |
| *Anaeromyxobacter* | -.429 | .289 | 8 |
| unidentified_Nitrospiraceae | .467 | .243 | 8 |
| *Stenotrophobacter* | .810^*^ | .015 | 8 |
| *Steroidobacter* | .217 | .606 | 8 |
| *Geobacter* | -.180 | .670 | 8 |
| *Desulfurispora* | -.905^**^ | .002 | 8 |
| Candidatus_Koribacter | -.571 | .139 | 8 |
| *Phenylobacterium* | .833^*^ | .010 | 8 |
| *Haliangium* | .108 | .799 | 8 |
| unidentified_Anaerolineae | -.357 | .385 | 8 |
| *Massilia* | .786^*^ | .021 | 8 |
| *Rubrivivax* | .491 | .217 | 8 |
| *Thermoanaerobaculum* | -.405 | .320 | 8 |
| *Azoarcus* | .563 | .146 | 8 |
| *Gracilibacter* | -.786^*^ | .021 | 8 |
| *Lentimicrobium* | -.405 | .320 | 8 |
| *Adhaeribacter* | .790^*^ | .020 | 8 |
| unidentified_Alphaproteobacteria | .310 | .456 | 8 |
| *Dongia* | .516 | .191 | 8 |
| *Microvirga* | .108 | .799 | 8 |
| *Acidibacter* | .833^*^ | .010 | 8 |
| *Gaiella* | .452 | .260 | 8 |
| *Vulgatibacter* | -.643 | .086 | 8 |
| *Woeseia* | .719^*^ | .045 | 8 |
| *Methylocaldum* | -.193 | .647 | 8 |
| Candidatus_Nitrososphaera | .171 | .686 | 8 |
| unidentified_Ruminococcaceae | -.262 | .531 | 8 |
| unidentified_Burkholderiaceae | .524 | .183 | 8 |
| *Skermanella* | .571 | .139 | 8 |
| *Altererythrobacter* | .371 | .365 | 8 |
| *Gemmatirosa* | .732^*^ | .039 | 8 |
| *Nocardia* | -.012 | .978 | 8 |
| *Nitrosospira* | -.554 | .154 | 8 |
| *Opitutus* | -.050 | .906 | 8 |
| unidentified_Nitrosomonadaceae | .275 | .509 | 8 |
| Candidatus_Entotheonella | .651 | .081 | 8 |
| unidentified_Clostridiales | .400 | .326 | 8 |
| *Lysobacter* | .542 | .165 | 8 |
| *Marmoricola* | .476 | .233 | 8 |

* represents a significant correlation (*p* ＜ 0.05), while ** represents a highly significant correlation (*p* ＜ 0.01).

**Table S4** HiSeq sequencing data of the metagenome.

| Sample | RawData | CleanData | Clean_Q30 | Number | N50 Len.(bp) |
| --- | --- | --- | --- | --- | --- |
| ANC | 6920 | 6892 | 93 | 193769 | 791 |
| ATC | 6786 | 6772 | 93 | 186023 | 955 |
| MTC | 6769 | 6732 | 94 | 231733 | 806 |

**Table S5** Physicochemical properties of the experimental soil.

| Index | pH | Electrical  conductivity  (μS⋅cm^-1^) | Total  nitrogen  (g⋅kg^-1^) | Total  phosphorus  (g⋅kg^-1^) | Organic  matter  (g⋅kg^-1^) |
| --- | --- | --- | --- | --- | --- |
| Values | 7.72 | 517.20 | 1.40 | 0.94 | 23.97 |

**Table S6 Experimental design.**

| Treatments | Labels | Mixing carbon fiber in soil (%) | Concentration (mg·kg^-1^) |
| --- | --- | --- | --- |
| Soil MFC spiked with tetracycline | MT | 1 | 10 |
| Anaerobic controls spiked with tetracycline | AT | 0 | 10 |
| Anaerobic controls without antibiotic | AN | 0 | 0 |


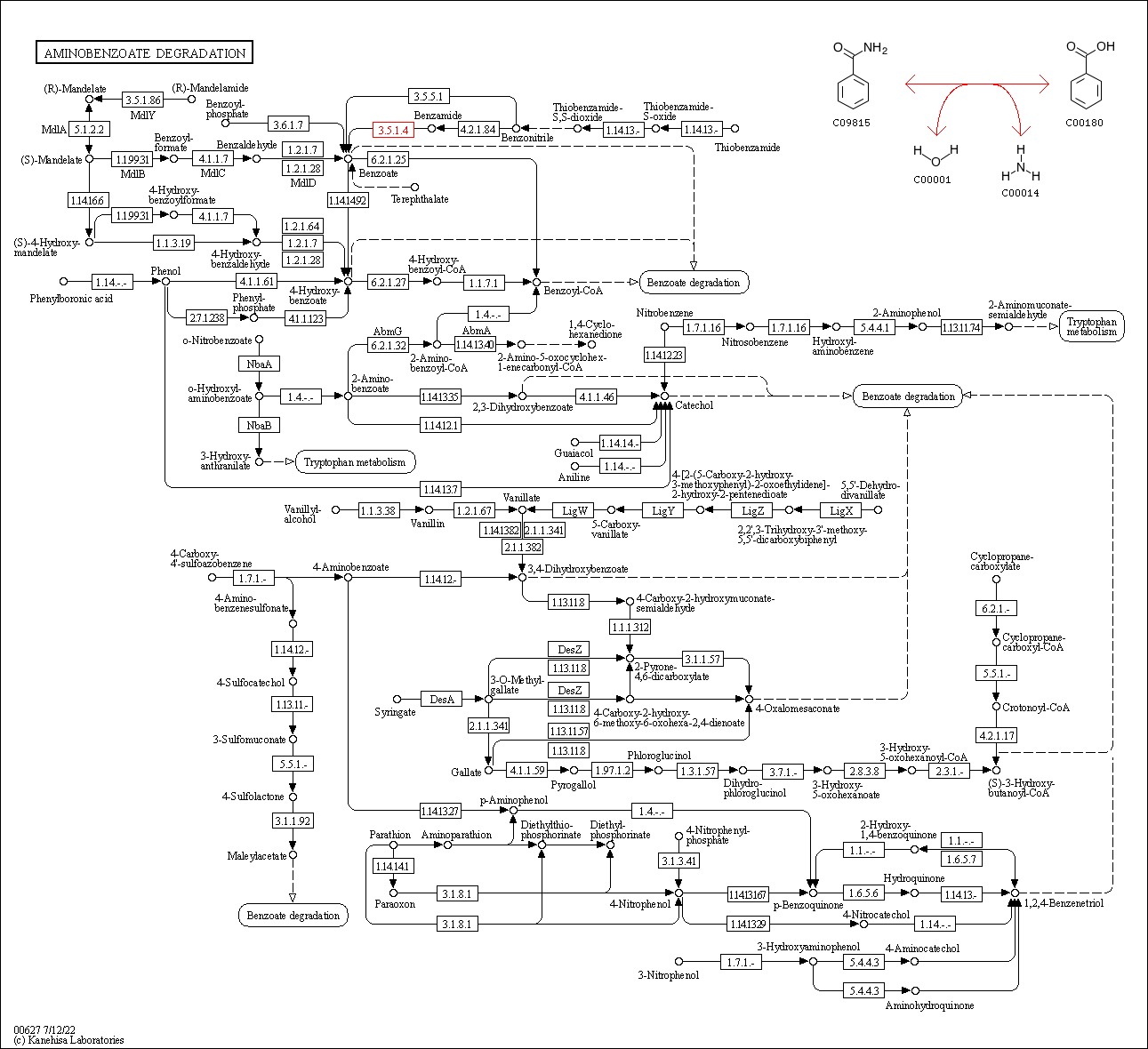


**Fig. S1.** Sketch map of KEGG metabolic pathways and related reaction equations (pathway: map00627, reaction: R05590).


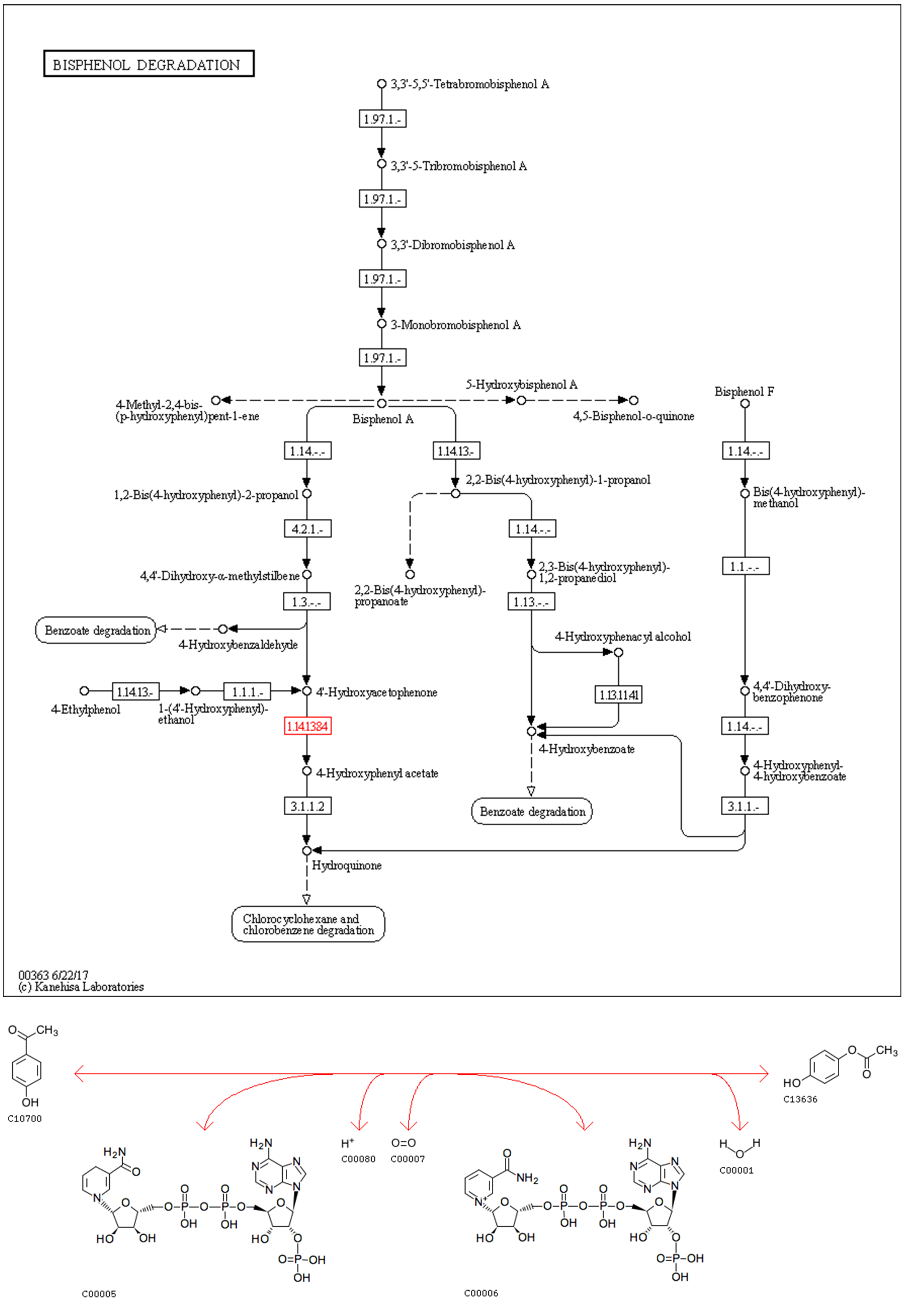


**Fig. S2.** Sketch map of KEGG metabolic pathways and related reaction equations (pathway: map00363, reaction: R06892).


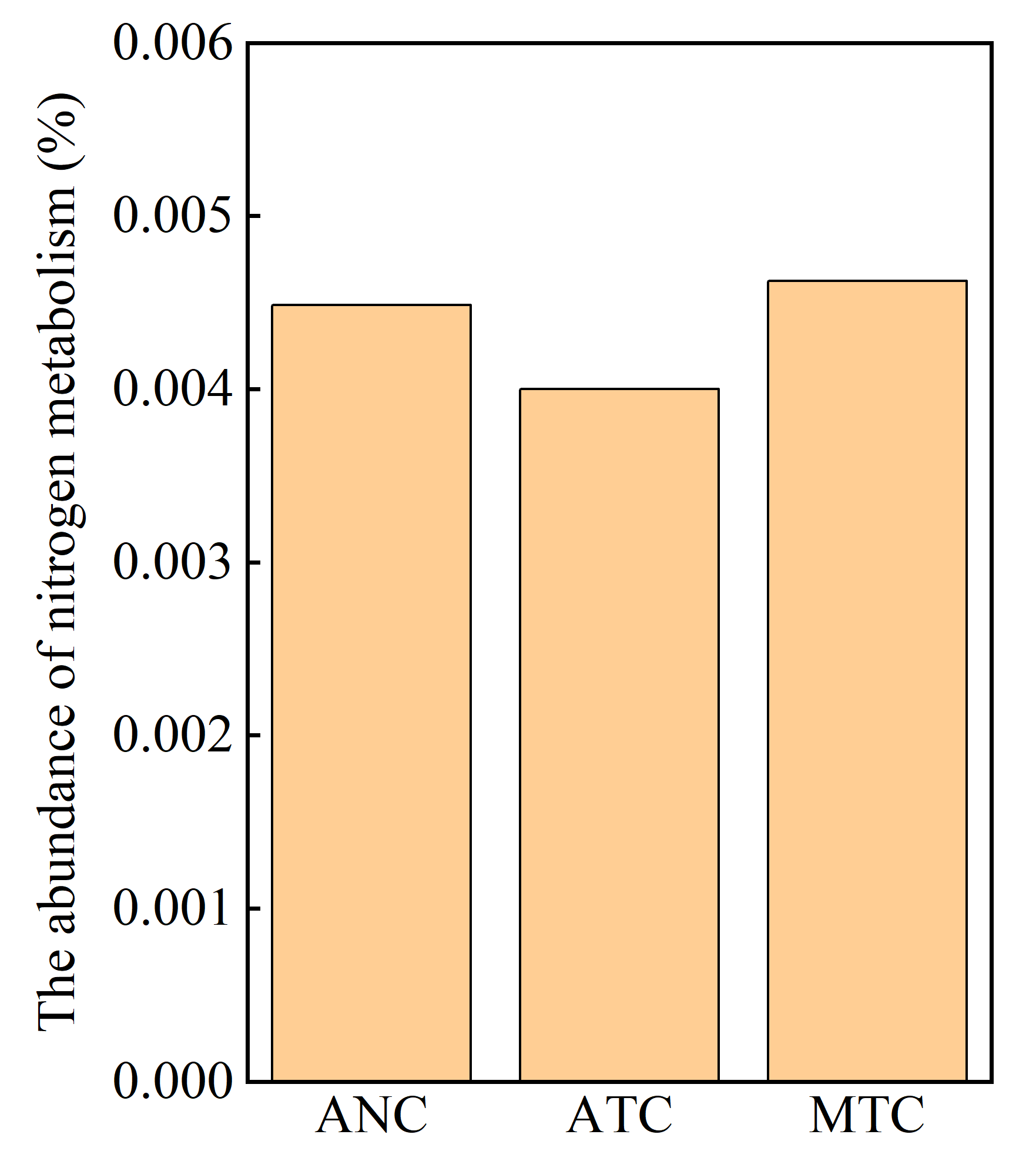


**Fig. S3.** The abundance of nitrogen metabolism in different treatments.


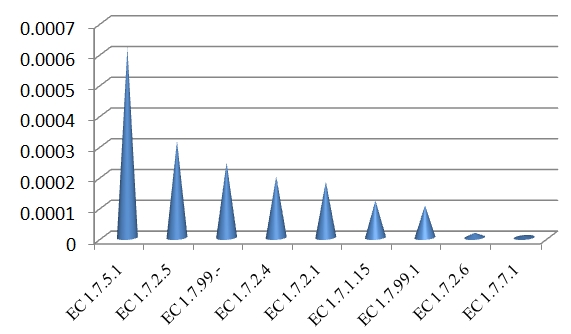


**Fig. S4.** The abundance of nitrogen cycling functional genes.


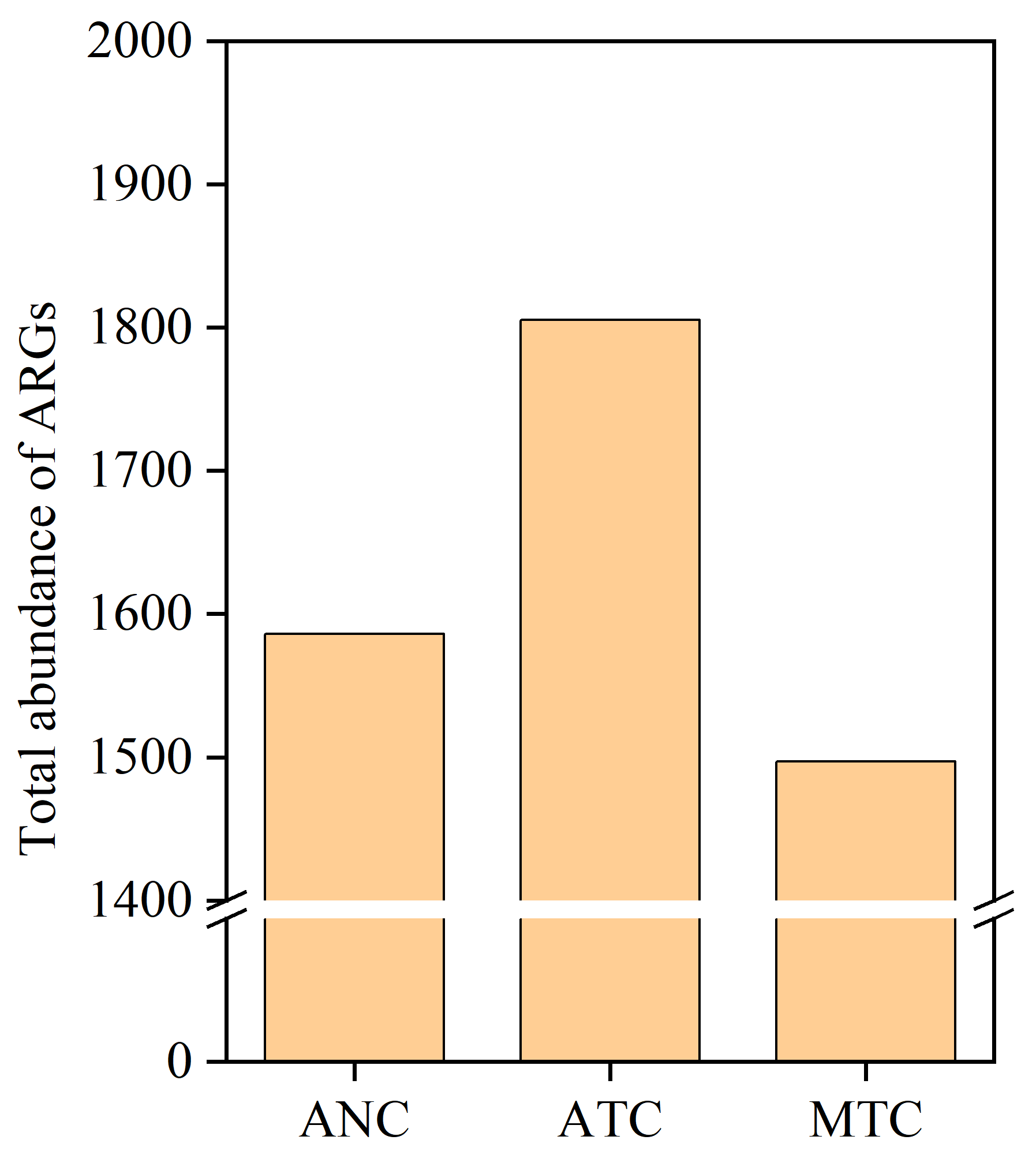


**Fig. S5.** Changes in the total abundance of ARGs in different treatments.


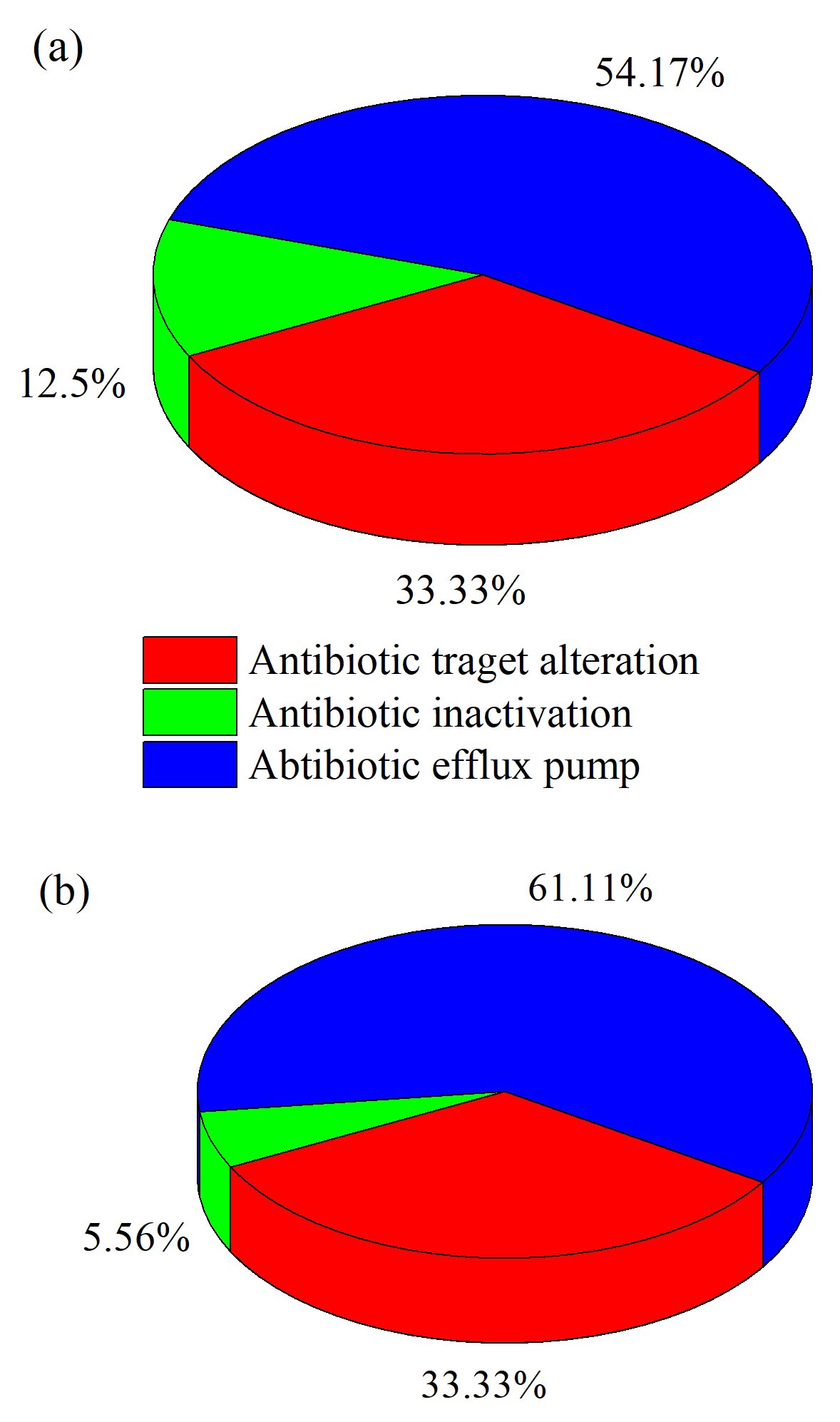


**Fig. S6.** The resistance mechanism of soil microorganisms to tetracycline in the ATC treatment (a) and the MTC treatment (b).
